# Application of Compressive Sensing to Ultrasound Images: A Review

**DOI:** 10.1155/2019/7861651

**Published:** 2019-11-15

**Authors:** Musyyab Yousufi, Muhammad Amir, Umer Javed, Muhammad Tayyib, Suheel Abdullah, Hayat Ullah, Ijaz Mansoor Qureshi, Khurram Saleem Alimgeer, Muhammad Waseem Akram, Khan Bahadar Khan

**Affiliations:** ^1^Faculty of Engineering and Technology, International Islamic University Islamabad, Islamabad 44000, Pakistan; ^2^Faculty of Electrical Engineering, Air University, Islamabad, Pakistan; ^3^Department of Electrical Engineering, COMSATS University, Islamabad, Pakistan; ^4^Institute of Fundamental and Frontier Sciences, University of Electronic Science and Technology, Chengdu, China; ^5^Department of Telecommunication Engineering, The Islamia University of Bahawalpur, Bahawalpur, Pakistan

## Abstract

Compressive sensing (CS) offers compression of data below the Nyquist rate, making it an attractive solution in the field of medical imaging, and has been extensively used for ultrasound (US) compression and sparse recovery. In practice, CS offers a reduction in data sensing, transmission, and storage. Compressive sensing relies on the sparsity of data; i.e., data should be sparse in original or in some transformed domain. A look at the literature reveals that rich variety of algorithms have been suggested to recover data using compressive sensing from far fewer samples accurately, but with tradeoffs for efficiency. This paper reviews a number of significant CS algorithms used to recover US images from the undersampled data along with the discussion of CS in 3D US images. In this paper, sparse recovery algorithms applied to US are classified in five groups. Algorithms in each group are discussed and summarized based on their unique technique, compression ratio, sparsifying transform, 3D ultrasound, and deep learning. Research gaps and future directions are also discussed in the conclusion of this paper. This study is aimed to be beneficial for young researchers intending to work in the area of CS and its applications, specifically to US.

## 1. Introduction

US imaging uses sound waves to produce images of the inside of the body. US is the most commonly used medical imaging modality for clinical diagnostics due to its diverse applications including cardiology, ophthalmology, pulmonology, nephrology, gynecology, urology, angiology, and general abdominal imaging [1–4]. US imaging is used as a guide for surgeries, small pieces of tissue examination, pediatric review, blood pressure, and blood flow direction estimation [5]. US imaging is the most often used modality by the physicians after radiography [4], as it is noninvasive, inexpensive, highly portable, and easily manipulable in medical diagnostics [6, 7]. It has strong connectivity and the ability to diagnose on-site in real time. US uses a variety of techniques for diagnosing various body parts and organs like Doppler ultrasonography, contrast ultrasonography, elastography, interventional ultrasonography, and compression ultrasonography. Several modes of US imaging can be used in many ways for medical diagnostics as discussed in [8–11].

The term ultrasound is used for frequencies above 20 KHz. Commercial ultrasonography imager uses ultrasound waves in the frequency range of 2–20 MHz. The important parameters which portray a wave are wavelength, frequency, intensity, and velocity. The velocity of the ultrasound waves in a medium is directly proportional to the medium stiffness and inversely to its density. Therefore, ultrasounds travel with high velocity in bones compared to water, fat, and tissue. The velocity of the US in bone, water, and fat is 4080 m/s, 1489 m/s, and 1450 m/s, respectively. US scanner works on principle of echo imaging. To generate US waves, electric current is applied to piezoelectric crystals, and the frequency generated by the crystal is directly proportional to the amplitude of the current. US transducers have an array of these elements often varying in number from 68 to 256. Frequency selection for scanning depends on the region of interest and technique used for diagnosis [12].

**Figure 1:**
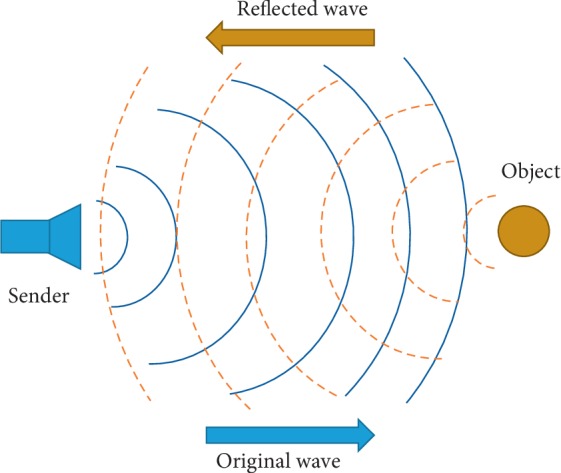
Basic working diagram of ultrasound.

For imaging, US transducer transmits the ultrasonic waves on region of interest to be scanned. Some of the US waves are absorbed by the subject body, while a large amount is reflected back towards the transducer. The reflected back waves are transformed to electrical signals by the transducer piezoelectric crystal and are further processed to form an image. Sampling of the reflected signals is done at Nyquist rate to avoid aliasing [13]. Current challenges in acquiring US images are high acquisition speed, high rate of sampling, analogue-to-digital converter, and faster processing units which are costly and consume more power. A basic working principle of US is shown in Figure 1.

## 2. Survey Methodology

To acquire scholarly work related to area of US image acquisition using CS, Google scholar, IEEE, Elsevier, Springer, and some other scientific search engines and research publishers were searched. The terms used for searches were compress sensing, compressive sensing, and compressed sensing along with the addition of ultrasound. More than ninety papers were acquired and studied for review, out of which twenty papers were selected for final draft. Papers obtained were from various conferences, letters, transactions, and journals from 2008 to 2018. Papers based on CS for Doppler US imaging, photoacoustic US imaging, or specific use of CS for US image noise removal, segmentation, classification, and dictionary learning are not in the scope of this work. Papers which used CS for US image acquisition and image reconstruction were selected for review. Five groups are made based on the objective of the paper; each group has four to five papers except group five which is based on deep neural networks and its application to US image reconstruction in our survey. Results of the papers are tabulated and discussed on the basis of evaluation metric of the algorithms. In the conclusion part of the paper, future directions on the basis of research gaps are also discussed.

## 3. Compressive Sensing

Compressive sensing (CS) is a method for recovering a compressed signal from far fewer samples than that needed in the Nyquist sampling model [14]. CS is dependent on three basic suppositions: the first one is the sparsity of original data or signal in a transform domain, the second is incoherence among sensing matrix (Ф) and transform matrix (Ψ), and the third is restricted isometric property (RIP). The number of samples needed to precisely reconstruct the compressed data is dependent on the specific reconstruction algorithm [14]. Almost all real world signals have the property of sparsity in one domain or another [15]. If the acquired data are not sparse in any transform domain, then the maximum number of data coefficients will be needed to reconstruct the data.

### 3.1. Sparsity

Most of the real world data/signals are sparse at least in some transform domain. For instance, image, seismic, and sound signals can be compressed and stored in terms of their projection using suitable model basis. When a model basis is selected suitably, the majority of the projection coefficients become very small or even zero and thus can be ignored. The definition of data sparsity can be mathematically interpreted as follows. Let a signal **x** *ɛ* *R*^*N*^, represented in a set of orthogonal basis [Ψ_i_]_*i*=1_^*N*^ such that **x** is a member of *R*^*N*^ and is shown in terms of *N* coefficients [*s*_*i*_]_*i*=1_^*N*^. The signal **x** can be specified as **x**= ∑_*i*=1_^*N*^*s*_*i*_Ψ_*i*_, where *s*_*i*_ symbolizes the coefficients of *N* × 1 vector *s* and  Ψ_*i*_ symbolizes the columns of matrix Ψ*N* × *N*. *s*_*i*_  are the coefficient sequences of *x* and *N* are the total coefficients of **x** [16]. In common, Ψ is named transform domain, sparsifying dictionary, or sparse domain [17, 18]. Signal **x** is mathematically stated as follows:

(1) $$\begin{array}{c} x=\Psi s. \end{array}$$

The signal **x** in equation (1) is *k*-sparse in transform basis Ψ if there exist **s** ϵ *R*^*N*^ which has only *K* nonzero coefficients. Commonly known compressible model bases are 2D wavelets, discrete cosine transform (DCT), discrete Fourier transform (DFT) to make images sparse, localized sinusoid to make audio signals sparse, curvelets for wave propagation, and fractal-type waveforms for spiky reflective data [19]. The recovery process of original vector **x** can be completed using the compressed vector **y** that has a dimension of *M* × 1 such that *M* < *N*:

(2) $$\begin{array}{c} y=\Phi x. \end{array}$$

The term [Φ_*i*_]_*i*=1_^*M*^ is an *M* × *N* matrix or group of vectors called measurement basis, measurement vectors, or sensing matrix with Φ_*i*_ representing rows. The value of **x** in equation (2) can be replaced with Ψ**s**, and we get

(3) $$\begin{array}{c} y=\Phi \Psi s, \end{array}$$

(4) $$\begin{array}{c} y=As, \end{array}$$

where **A** in equation (4) is a matrix of *M* × *N* dimension and is helpful in transformation of vector **x** that is a *k*-sparse signal into **y** measurements of *M* × 1.

### 3.2. Incoherence

Coherence is the measure of correlation between the elements of two matrices. The matrices could be in dissimilar representation domain of different dimensions. For example, we have Ψ matrix of *N* × N dimension with Ψ_1_, Ψ_2_,…, Ψ_*N*_ as its column vectors and Φ as an *M* × *N* matrix having Φ_1_, Φ_2_,…, Φ_*M*_ rows. In equation (5) *μ* represents coherence and is mathematically stated as

(5) $$\begin{array}{c} µ\;\left(\Phi ,\;\Psi \right)\;=\sqrt{n\;\cdot \max\langle \Phi_{p}\;,\;\Psi_{o}\rangle }, \end{array}$$

where 1 ≤ *o* ≤ *N* and 1 ≤ *p* ≤ *M*. Thus, following the principle of Linear Algebra, we get

(6) $$\begin{array}{c} 1\;\leq \;µ\;\left(\Phi ,\;\Psi \right)\;\leq \sqrt{N}. \end{array}$$

In framework of CS, our main objective is to get maximum incoherence between the matrix utilized for sensing, sampling, and compression of data (Φ) and the matrix used for representation of signal as sparse (Ψ) [19]. The more *µ*(Φ,  Ψ) is near to 1, the more the incoherence between Φ and Ψ basis is.

### 3.3. Restricted Isometry Property (RIP)

The idea of RIP was first presented in [20] and has been used to solve many theorems in CS [21]. RIP characterizes matrices which are almost orthonormal, at least when in use on sparse vectors. An important consideration of compressive sampling is that the information of signal must be preserved by measurement matrix which is guaranteed by checking the RIP of measurement matrix Φ. RIP is defined on isometry constant *δ*_2*k*_ of a matrix, which is the smallest number such that equation (7) holds for all *k*-sparse vectors *x*. Similarly, if *δ*_2*k*_ is smaller than one, then all pairwise distances between *k*-sparse signals must be well kept in the measurement space, as shown in equation (7) for *k*-sparse vectors *x*_1_ and *x*_2_:

(7) $$\begin{array}{c} \left(1-\delta_{2k}\right){\left\|x_{1}-x_{2}\right\|}_{2}^{2}\leq {\left\|\Phi x_{1}-\Phi x_{2}\right\|}_{2}^{2}\leq \left(1+\delta_{2k}\right){\left\|x_{1}-x_{2}\right\|}_{2}^{2}. \end{array}$$

### 3.4. Compressive Sensing in Ultrasound

CS has the ability to reconstruct the images and signals from far fewer samples required in traditionally used Shannon Nyquist sampling theorem. The main concern of CS is that the data must be sparse in some model basis, e.g., Fourier basis, wavelet basis, and dictionary learned from data. US is a medical imaging modality which acquires image by the beam forming of the set of raw RF signals received at each transducer element. CS in ultrasound reduces the number of transducer elements and thus reduces acquisition time and power consumption.

## 4. Reconstruction Model

For CS reconstruction nonlinear algorithms are used, which requires information of sparsifying matrix, where data are sparse or compressible. In equation (4), *s* is a *k*-sparse vector representing projection coefficients of **x** on **s**_**i**_, **y** is the measurement vector, and **A** is the MxN matrix (**A**=ΦΨ), where **s** is the original ultrasound signal vector and **x** is the sparse representation of that vector. To make signal or vector *s* sparse Ψ is used, Φ is the measurement matrix used to sample and compress the vector **x**, and **y** is the compressed signal called measurement vector obtained from the product of matrix **A** and ultrasound signal vector **x**. To solve and prove that solution of equation (8) is sparse reconstruction, *L*_0_, *L*_1_, and *L*_2_ norms are used over solution space [22]. Using least square solution which is the minimization of *L*_2_ norm, the solution can be presented as

(8) $$\begin{array}{c} x=\text{min}\left\|x_{2}\right\|=A^{\text{T}}{\left(AA^{\text{T}}\right)}^{-1}Y, \end{array}$$

(9) $$\begin{array}{c} \left\|x_{2}\right\|=\sqrt{\left(\overset{N}{\underset{i=1}{\sum }}x_{i}^{2}\right)}. \end{array}$$

Using *L*_1_ minimization matching pursuit (MP) or basis pursuit (BP) etc., the signal is accurately reconstructed from vector **y** having *M* number of compressed measurements by solving a simple convex optimization problem [23]. Reconstruction of signal using *L*_1_ minimization can be stated as

(10) $$\begin{array}{c} x=\text{min}{\left\|x_{1}\right\|}_{1}, \end{array}$$

(11) $$\begin{array}{c} \left\|x_{1}\right\|=\sqrt{\left(\overset{N}{\underset{i=1}{\sum }}\left|x_{i}\right|\right)}. \end{array}$$

Using *L*_2_ minimization for nonsparse signals gives disappointing results [21]. As most of real world signals are not sparse, therefore, *L*_2_ minimization is an inappropriate choice for signal reconstruction. In most of the cases, *L*_0_ minimization guaranties perfect results although that involves more computation. Under certain conditions *L*_1_ norm gives same results as *L*_0_ [24, 25].

## 5. Performance Evaluation Parameters

For the evaluation of reconstructed images, various evaluation parameters have been used by researchers for the comparison of their results. The most common evaluation parameters used are signal-to-noise ratio (SNR), contrast-to-noise ratio (CNR), mean square error (MSE), root mean square error (RMSE), normalized root mean square error (NRMSE), peak signal-to-noise ratio (PSNR), structural similarity index (SSIM), and mean absolute error (MAE). Some of them are discussed below.

### 5.1. Mean Absolute Error (MAE)

The accuracy of signal or data after CS reconstruction is obtained by comparing original data or image with reconstructed data or image using

(12) $$\begin{array}{c} \text{MAE}=\frac{1}{N}\left(\overset{N}{\underset{i=1}{\sum }}\left|I_{oi}-I_{ri}\right|\right), \end{array}$$

where *N* represents the total number coefficients of image data, *I*_*oi*_ is the intensity or amplitude of the *i*^th^ original image pixel, and *I*_*ri*_ is the intensity or amplitude of the *i*^th^ processed image pixel.

### 5.2. Signal-to-Noise Ratio (SNR)

SNR is used to evaluate the image quality with the definition as

(13) $$\begin{array}{c} \text{SNR}=10\;{\text{log}}_{10}\left[\frac{\mu }{\delta }\right], \end{array}$$

where *μ* denotes the mean and *δ* represents the standard deviation of noise.

### 5.3. Root Mean Square Error (RMSE)

RMSE is a quadratic scoring rule that gives the average of the error. RMSE is used to measure accuracy for continuous variables. In equation (14), I_S_ is the intensity of original image pixel and *I*_*ri*_ is the intensity of processed image pixels. Mathematically, RMSE is written as follows:

(14) $$\begin{array}{c} \text{RMSE}=\sqrt{\frac{1}{N}\left(\overset{N}{\underset{i=1}{\sum }}{\left|I_{S}-I_{ri}\right|}^{2}\right)}. \end{array}$$

### 5.4. Structure Similarity Index (SSIM)

SSIM is a measure of the degradation originated because of data compression or due to data losses during transmission. Two images are needed for SSIM calculation: original image and the reconstructed one. SSIM is mathematically expressed as

(15) $$\begin{array}{c} \text{SSIM}\left(I,o\right)=\frac{\left(2\mu_{I}\mu_{o}+C_{1}\right)\left(2\sigma_{Io}+C_{2}\right)}{\left(\mu_{I}^{2}+\mu_{o}^{2}+C_{1}\right)\left(\sigma_{I}^{2}+\sigma_{o}^{2}+C_{2}\right)}, \end{array}$$

where *σ*_*I*_ characterizes the standard deviation and *μ*_*I*_ expresses the average value of original image *I*. The terms *σ*_0_ and *μ*_0_ represent standard deviation and average value of the processed image. Here in equation (15), *σ*_*Io*_ express the correlation coefficients of the original and processed images. *C*_1_ and *C*_2_ stabilize the division with a weak denominator [26]. If SSIM results in the value 0, then the images are totally dissimilar, and two images are completely matched if the value is 1.

### 5.5. Contrast-to-Noise Ratio (CNR)

CNR determines the quality of reconstructed image in comparison to original image. CNR is similar to the SNR with the difference that it subtracts some image intensity term and then calculates the ratio [27]. CNR is given by [28] as

(16) $$\begin{array}{c} \text{CNR}=\frac{\left|S_{O}-S_{P}\right|}{\sigma_{0}}, \end{array}$$

where *S*_*O*_ represents the image intensity of producing structure *O*, *S*_*P*_ denotes the image intensity for image producing structures *P* in the region of interest, and *σ*_0_ represents the standard deviation of the original image noise.

## 6. Classification of CS Algorithms in Ultrasound

**Figure 2:**
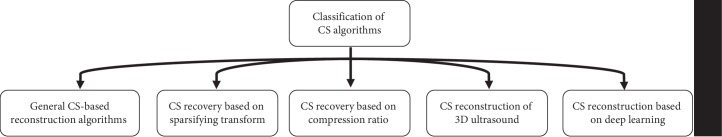
Classification of various CS reconstruction algorithms.

There has been a lot of work done on different aspects of medical ultrasound. Researchers are working to improve the CS reconstruction techniques for achieving good quality images with high compression ratios and greater accuracy. In this section, groups are made on the basis of various algorithms and reconstruction techniques, keeping in view their different attributes. The classification of CS algorithms is shown in Figure 2.

### 6.1. General CS-Based Reconstruction Algorithms

A number of algorithms have been used for CS, where each gives unique results for the reconstruction accuracy and computation time. This group consists of algorithms which are used for US acquisition or reconstruction.

Donoho introduced the idea of approximate message passing (AMP) in CS [29]. AMP is used for image reconstruction in compressive sampling framework with various denoising algorithms [30, 31]. Kim in [32] used AMP as a CS reconstruction algorithm for US image reconstruction. To achieve better image quality and compression ratio, several combinations of denoiser and transform domains are used. Amplitude scale invariant Bayes estimator (ABE) and soft thresholding (ST) are used as denoiser in spatial domain, wavelet domain, and DCT. The steps of AMP iteration are defined in

(17) $$\begin{array}{c} x^{t+1}=\eta_{t}\left(A^{*}z^{t}+x^{t}\right), \end{array}$$

(18) $$\begin{array}{c} z^{t}=y-Ax^{t}+\frac{1}{\delta }z^{t-1}\langle \eta_{t-1}^{\prime }\left(A^{*}z^{t-1}+x^{t-1}\right)\rangle . \end{array}$$

where **x**^*t*^ is an estimate of **x** at *t*^th^ iteration, *η*_*t*_ is the thresholding function derivative, **A**^*∗*^ is the transpose of measurement matrix, *z*^*t*^ represent the error, and *δ* is the measurement rate.

Hill in [33] has used AMP with image denoiser, which is based on a heavy tailed distribution. AMP has been used specifically as a Cauchy prior based maximum a posteriori (MAP) approximation within a wavelet-based compressive sensing pattern. The MAP denoising algorithm results in very fast convergence, which is approximately twice fast in comparison to the AMP method for image compressive sensing. To benchmark the performance of the proposed system, the authors have proposed two other methods: amplitude scale invariant Bayes estimator and soft thresholding.

Achim [34] extended their previous proposed approach for CS reconstruction of RF US images. This approach of RF signal reconstruction has used symmetric alpha-stable-IRLS (S*α*S-IRLS) algorithm in the Fourier domain with prior information about US RF signals. The first information based on observation is that US RFs are best characterized by statistical use of alpha-stable distribution, and the second one is that RF echoes are easily assumed in Fourier domain. Using these two observations, IRLS has been used. Through simulation, it is proved that RF echoes give best results using the proposed *L*_*P*_-norm minimization with the two prior observations. Reconstruction is done using the proposed *L*_*P*_ minimization method. SSIM and NRMSE results show that the proposed S*α*S-IRLS-DP gives better results in comparison to S*α*S-IRLS and S*α*S-IRLS-FD.

Liu [35] proposed unique CS-based synthetic transmit aperture (STA) technique for achieving a high-frame-rate, high-contrast, and high-resolution US imaging. In STA, one element of US transducer transmits plane wave sequentially at a time with random apodization for several times and all the elements receive the corresponding echoes. CS-STA uses a less number of plane wave firings than STA. A reconstruction algorithm is then applied on CS-STA to recover full dataset of STA from recorded echoes of CS-STA. A B-mode image is formed from the data by applying the SA beam-forming algorithm. The advantage of CS-STA is the use of less ultrasound firings than STA firing to achieve high frame rates. CS-STA also maintains high resolution, as full dataset of STA is recovered by CS and due to plane wave firing the contrast is improved. **y** is a sparse signal having most of its entries zero. Authors have solved the problem using convex optimization as shown in

(19) $$\begin{array}{c} v=\;\frac{\text{arg min}}{v\in R^{n}}\;{\left\|v\right\|}_{1}\text{subjected to }{\left\|y-Av\right\|}_{2}\leq \varepsilon . \end{array}$$

Quinsac [36] has presented a Bayesian reconstruction framework for CS of ultrasound images using frequency domain as sparsifying basis. Bernoulli Gaussian prior is applied to the DCT of US images for achieving sparsity. To reconstruct US image Bayesian CS is used by the authors in [35]. The Bayesian method allows the image sparse level to be estimated in a spectral domain. Furthermore, it is a useful parameter in *L*_1_ constrained minimization problem. The advantage of the proposed method is the increase in frame rate acquisition. Moreover, it is completely automatic and no requirements are needed to adjust parameters. For comparison, US image is reconstructed using classical techniques and proposed Bayesian based algorithm. Achim [36] first time proposed iteratively reweighted least squares algorithm (IRLS) algorithm to US RF signal for *L*_P_-norm minimization. All the above discussed CS algorithms along with their results are summarized in Table 1.

### 6.2. CS Reconstruction Algorithms Based on Sparsifying Transforms

Every real world signal is sparse in some specific transform domain and if right sparsifying transform is selected for a signal, one can get data or image with improved results for a large compression ratio. In 2010 Friboulet [37], used directional wave atom as model basis for reconstruction, Fourier model basis and daubechies wavelet model basis were used for the comparison. The obtained results shows that directional wave atom basis gives better results in comparison to others two model basis at a sampling rate of 50% to 90%. Reconstruction was done by solving *L*_1_ minimization using *L*_1_-Magic-Packet. The accuracy was given by comparing the results of CS reconstruction with the original signal data using MAE. In [38] Chu has used DCT and discrete wavelet transform (DWT) as model basis. They have discussed CS reconstruction performance of three different types of US post-beam-formed data. The three post-beam-formed data types reconstructed are radio frequency (RF), envelope, and log converted data. Individually data type has a unique signal spreading and thus has unique sparse representation which affects sparsifying effectiveness in the process of CS reconstruction and quality of reconstructed images. DWT was used for experiment, and MSE results of US image data have shown that CS reconstruction of post-beam-formed data has the minimum value of error. Results of DCT as a sparsifying domain were the same as DWT; therefore, they were not discussed in their paper.

Kumar [40] discussed frequency and time domain beam-formed matrices and their implementation as CS matrix for US image reconstruction. Using beam-forming matrices, a direct US image reconstruction based on CS is presented. The authors in [34] developed an ultrasound time domain model for beam forming along with a frequency domain equivalent. In CS sparse recovery, they used this model based on matrices of time and frequency domain to recover images from undersampled ultrasound waves. Time domain ultrasound beam-formed matrices are given by

(20) $$\begin{array}{c} K=\left[\begin{array}{c} A\left(i,\;j,1\right)\\ \vdots \\ A\left(i,j,N_{0}\right) \end{array}\right]. \end{array}$$

Frequency domain US beam-formed matrix is represented by

(21) $$\begin{array}{c} K^{\prime }=\left[\begin{array}{c} B\left(i,j,1\right)\\ \vdots \\ B\left(i,j,N_{0}\right) \end{array}\right]. \end{array}$$

The beam-forming matrices use the Fourier transform as a sparsifying domain. The CS matrix is created by taking the product of beam-forming matrix with Fourier matrix. The image is recovered directly from the revived signals using orthogonal matching pursuit (OMP) for recovery. The experiments were done with half of the minimum needed sampling rate.

Kim [32] used CS reconstruction algorithm AMP for US image reconstruction and spatial or time domain, wavelet domain, and DCT as sparsifying domains. AMP is an iterative algorithm which reconstructs the image by compressive sampling and image denoising. ABE and ST are used as denoiser. PSNR and SSIM results show that DCT with ABE denoiser has better results. In 2017, Shin [39] used CS applied to ultrasound elastography. Their research has used three different model bases or sparsifying matrices, i.e., DCT, WA, and DFT, and two recovery frameworks, i.e., block sparse Bayesian learning (BSBL) and *L*_1_ minimization. Results of sparsifying model basis and reconstruction algorithm were compared. They have suggested that DFT for sparsifying and BSBL algorithm for reconstruction has the best results at 60% compression. The reconstruction of the RF ultrasound sampled data is done by solving the following *L*_1_ minimization problem:

(22) $$\begin{array}{c} \left(x^{k+1},a^{k+1}\right)=\;\frac{\text{arg min}}{v\in R^{n}}\;{\left\|v\right\|}_{\text{l}1}\text{ subjected to }{\left\|y-A^{\text{cs}}v\right\|}_{\text{l}2}\leq \epsilon . \end{array}$$

Comparison of mentioned techniques and their results is given in Table 2.

### 6.3. CS Reconstruction Algorithms Based on Compression Ratio

CS is used to decrease the number of samples during acquisition or reconstruction of some data from a minimum number of samples. In this group, those research papers which have used CS and achieved good reconstruction results for large compression ratios up to 80% are discussed. Results are given in Table 3.

Zobly in [41] proposed a novel structure of CS sampling theory for Doppler US signal reconstruction. OMP and compressive sampling matching pursuit (CoSaMP) have been used in their study. OMP algorithm is less complex and fast for recovering high-dimensional sparse signals in comparison to CoSaMP. In their experiments, both algorithms recovered images in a short period of time with sufficient accuracy from a minimum number of samples. Results show that OMP performs faster than CoSaMP. However, CoSaMP has better reconstructed image quality in comparison to OMP.

In 2015, Chen [42] used novel framework of compressive deconvolution (CD) for reconstructing enhanced RF images from measurement vector. CS and deconvolution are combined and applied to US imaging, which resulted in compressive deconvolution model. This technique helps to reduce US data volume and improve image quality. Solution of proposed compressive deconvolution is centered on alternating direction method of multipliers (ADMM) that utilizes two constraints. The first constraint is image sparsity in some sparsifying domain like Fourier, wavelet, etc. The other constraint is the prior information for the tissue reflective function (TRF). For sparsifying transform generalized Gaussian distribution (GGD) model is used. The authors experimented the algorithm for the reconstruction on the modified Shepp-Logan phantom with 20–80% samples removed.

Chen [45] worked on compressive deconvolution for US imaging using supposition of GGD TRF. Their work focuses on application framework of CD on US imaging and suggested an updated *L*_*p*_-norm algorithm based on ADMM. The solution is based on ADMM which utilizes two constraints, i.e., sparsity of the RF images in the 2D Fourier domain, and the *L*_*P*_ norm, i.e., the norm used to recover the images [46]. The main purpose of their work was to approximate the TRF **x** from the fuzzy and compressed measurements **y**. Based on the supposition of GGD **x**, TRF's *L*_*P*_-norm minimization is utilized. The results confirmed the dominance of proposed simultaneous CD method that has better performance than the sequential approach.

In 2017, Chen [43] applied CD framework to estimate point spread function (PSF). The model of compressive deconvolution uses 2D convolution operator, which has the information on the system PSF with a measurement matrix. The author has proposed a unique alternating minimization-based optimization method, which inverts the resulting linear model. This method results in enhanced US image and simultaneously gives the estimation of the PSF. Chen [44] used CD and achieved US data from a minimum number of samples for US image and improvement in image spatial resolution. In this research, previous work of the CD is extended and is aimed to jointly estimate system PSF and US image. For a known PSF (estimated in [42]), they proposed compressive semiblind deconvolution (CSBD) which has proved the capability to recover improved ultrasound images from compressed measurements by reversing forward linear model. To get quantitative results of the proposed algorithm, the authors have first used the restoration of TRF for RF images and then PSF for simulated data.

With proposed CSBD, two other CS deconvolution algorithms, CD and CD_true, were also implemented by the authors and the results were compared. All the mentioned algorithms and their results are summarized in Table 3.

### 6.4. CS Reconstruction of 3D Ultrasound

Not much research has been done in the field of 3D US compressive sensing. Research work in this field has mainly two directions, 3D US image CS acquisition and 3D US image reconstruction. In this section, we have included both mentioned categories. CS framework was first adapted to 3D ultrasound in 2010 by Basarab [47]. They used three different undersampling patterns and a nonlinear conjugate gradient algorithm for reconstruction. The US images are reconstructed in the Fourier domain as CS gives best results when original data samples are linear combinations of the data to be reconstructed. The results obtained from NRMSE between original image and reconstructed image from 50% samples of the original data show minimum data loss. To recover original *K*-space data, optimization routine is used as follows:

(23) $$\begin{array}{c} \frac{\text{arg min}}{M}{\left\|\mathrm{AM}-y\right\|}_{2}+\lambda {\left\|\Psi M\right\|}_{1}. \end{array}$$

3D volume of consecutive RF images in *K*-space is represented by *M*=Fm, sampling scheme is denoted by *A*=Φ*F*^−1^, *F*^−1^ represents the inverse Fourier transform and Φ are the RF random sample locations, *y* denotes the RF ultrasound image compressed measurements, and Ψ denotes the sparsifying model basis and the coefficients weights for sparsity in model basis. Three unique sampling schemes Φ_1_, Φ_2_, and Φ_3_ are represented and evaluated in their research. Φ_1_ is a uniform random sampling pattern in three directions and Φ_2_ is proposed to reduce the number of US pulse emissions. To make US image *K*-space sparse, three sparsifier finite differences, discrete cosine, and daubechies wavelet transforms were used on the simulated RF images in frequency domain.

In [48], the authors have proposed a new approach for 3D ultrasound compressive sampling, based on learned overcomplete dictionaries. These dictionaries represent a signal sparse as these dictionaries are designed for a particular category of images. Two undersampling patterns for the 3D US imaging are experimented by them. The first one is a spatially uniform random acquisition and the second one is a line-wise random acquisition. K-SVD algorithm is used for dictionary learning on patches extracted from the training dataset. K-SVD learned dictionaries have shown a minimal information loss in comparison to fixed sparsifying transform indicating the efficiency of overcomplete dictionaries. In 2015, Lorintiu [49] used K-SVD with the improvements proposed by [51]. K-SVD algorithm is used for dictionary learning. On-log envelope data are utilized, by removing 20%–80% of the original acquired data as in [48].

The influence of the sampling strategy and training parameters are evaluated on simulated images. Comparison results of two sampling patterns, which were line-wise and point-wise, have shown that line-wise sampling pattern is more accurate, making CS acquisitions of 3D data possible. CS acquisition of 3D data offers increased frame rate by leaving out the acquisition of RF lines. The method is applied to several in vivo and ex vivo organs of US volumes. It was shown that overcomplete dictionaries have better performance in comparison to Fourier, Cosine, or other fixed transforms. Finally, the generality of learned dictionary approach is investigated with the possibility of building a general dictionary, which can be used to reconstruct different in vivo and ex vivo organs of different volumes reliably.

In 2017, Kruizinga [50] introduced an US transducer of one element with a plastic aperture mask to acquire a 3D US image. They used a plastic mask with a different coding in front of its aperture. This mask acts as a distorter (to RF reflected back signals) by inducing varying echo delays and guarantees that pixels in the image are independently identifiable in the measurement vector. The result is a compression of spatial US field on the transducer surface having enough information to make a 3D image. Using a single sensor transducer, measurements are obtained by rotating a plastic mask at 180° in front of transducer to maximize the condition of the reconstruction problem. An approximate signal model that captures the ultrasonic response of the mask and uses it to pose mask shape optimization as a sensor selection problem is defined. This is solved by converting it to a convex problem using a greedy selection method. Algorithms discussed in this section are summarized in Table 4.

### 6.5. CS-Based Deep Learning Novel Methods

The architecture of deep neural networks (DNNs) had been very useful by performing various artificial intelligence tasks. It is applied to US imaging in liver classification [52], locating standard plane in fetal US imaging [53], and classification of breast lesions [54]. In this group, a research work using DNN and CS for US image reconstruction is reviewed. Other papers added in the group are using CS and DNN for magnetic resonance image (MRI) recovery, as our review paper is not discussing MRI but the techniques/algorithms can be used and shall be helpful for US images recovery.

A novel framework using DNN for the compression and decompression of US signals based on stacked denoising autoencoders (SDAs) is proposed in [55]. The framework consists of four layers: the first layer is responsible for the compression of the signal and remaining hidden and output layers are applied for the reconstruction of the signal. They have investigated a linear measurement case (SDA-L), where the compression matrix is not learned, and also a nonlinear measurement case (SDA-NL), where the compression is indicated as a layer of the network. The publically available dataset PICUM is used to evaluate the performance of the network; the network is trained using simulated US signals. They have obtained and compared the PSNR results of the proposed SDA-NL, SDA-L, and CS algorithms, which indicated that the SDA-CL has better results than SDA-CNL and CS. The results of the proposed frameworks in the table are obtained using longitudinal common carotid images from the mentioned dataset.

In 2016, Sun [56] introduced a novel deep learning framework ADMM-net to get better reconstruction results. ADMM-net is defined on a dataflow graph, derived from the iterative process in ADMM used for the optimization of CS-based models. Limited memory Broyden–Fletcher–Goldfarb–Shanno (L-BFGS) algorithms are used to train all the parameters of the network, i.e., image transforms, shrinkage function, etc. The ADMM-net uses optimized parameters, which are learned from the training data for CS-based reconstruction task. The reconstruction results using different compression ratios are given in Table 5.

Wen et al. [58] proposed a robust CS method for sparse reconstruction, using a first order algorithm based on ADMM. The ADMM is not convergent for nonconvex problems, and *L*_1_ loss is used for convergence and as a smoothing operator. *L*_1_ norm is used as a loss function for the remaining error and uses generalized nonconvex penalty for sparsity. The *L*_1_ loss is more protective to the outliers in the measurements than *L*_2_ loss, and due to the nonconvex nature, more accurate recovery can be obtained. ADMM is proposed to solve the nonconvex and nonsmooth minimization problem. To make the algorithm convergent, a smoothing technique has been applied on *L*_1_ loss function and an adequate condition for the convergence of proposed algorithm has been made available for nonconvex regularization.

In 2016, Eksioglu [59] introduced a novel approach for the reconstruction of magnetic resonance (MR) images utilizing nonlocal block matching (BM3D) image model. The modified BM3D-MRI consists of fully decoupled observation fidelity and model fidelity steps. The decoupling allows for the adoption of a varying regularization parameter strategy, enhancing the performance. The final algorithm is very easy to use and understood, using only three parameters for tuning. The reconstruction results of BM3D are given in Table 6 for comparison.

In 2018, Li [60] used a novel composite robust ADMM (Co-Robust-ADMM) for reconstruction. S*α*S is a usual impulsive noise commonly formed during signal acquisition or transmission. Robust CS is a method of signal reconstruction from a minor number of undersampled data in the existence of impulsive noise. A smoothing strategy is utilized to counter the effect of *L*_1_ norm, as it has a nonsmooth optimization nature. They have trained DNN using a model of robust composite regularization to make use of more previous knowledge and image features. The DNN is employed to train the samples for the best possible parameters, shrinkage function and the transform domain, and can be kept as a network. The acquired network is utilized for refining recovery results in which DCT is used as an initial sparsifying transform. The comparison of the proposed framework is done with other existing robust CS methods using PSNR; it has proven that the proposed algorithm results are much better. The authors used 5 MR images with impulsive noise (*α* = 1.0, *γ* = 10–4) for network training and for the reconstruction of brain images with different noise levels. For comparison, we have shown their results of the offered algorithm in Table 5 with only one noise level, i.e., *α* = 1.0 and *γ* = 10–3.

## 7. Datasets

In this section, several US image datasets are discussed. Normally, the researchers test their proposed algorithms on simulated datasets followed by real US datasets. The most commonly used simulation software for US images generation is Field II [61]. This software can simulate US sample images, cyst images, or images mimicking human or animal organs. For simulation purpose, the size of the image plays important role; increasing or decreasing its size is somehow related to image recovery, quality, and algorithm execution time. Another important consideration for US image acquisition is the selection of scanning probe, sampling frequency, central frequency, sampling axis, and pitch. Field II can model the scanning probe, including probe voltage, number of US elements, scanning depth, sampling frequency, central frequency, sampling axis, pitch, scanning frequency, and frame rate. Datasets are mainly divided into two categories: (i) real acquired US images and (ii) simulated US images.

### 7.1. Real Images Datasets

Normally, US images are acquired on demand as per researcher requirement. However, there are some online databases which can be accessed for validating the results of the proposed algorithms. In this research, various real US images and datasets were observed including human thyroid, kidney, cardiac and liver images, mouse liver, kidney, and embryo images. In [36], real US images are acquired using US scanner for CS. The most commonly used US scanner for acquiring images was clinical scanner Sonoline Elegra, which was modified for research with linear probe (Siemens Medical Systems, Issaquah, WA, USA) [32, 34, 36]. The sampling frequency and central frequency are also changed in some research studies, in which RF US tissue images are commonly obtained with central frequency of 7.5 MHz and sampling frequency of 50 MHz and a real human right lobe thyroid gland image with sampling frequency adjusted to 50 MHz and 40 MHz [32, 34, 36]. In [34], an image of real human liver is acquired for the CS reconstruction using the same settings.

In [47], the authors used a SHERPA high-resolution single element probe with frame rate of 10 images per second. The probe frequency was 2 MHz; sampling frequency, 80 MHz; scanning frequency, 20 MHz; scanning width, 16 mm; and exploration depth, 2.8 mm. The acquired image was an in vivo anaesthetized mice embryo. In [42], real images of a mouse kidney and bladder were acquired using a single element probe. Also in [44] an image (size 250 × 180) of real mouse kidney was acquired using a single element US probe.

In [33], a dataset used for US recovery, which is available online at http://research.microsoft.com/enus/projects/objectclassrecognition. The dataset is known as “pixel-wise labelled image database v2” and consists of 591 US images. In [41], the experiments were completed using Doppler US imaging spectrum data. The data are available online at Torpp group websites having a total length of 2032.

In [50], 3D scanning of objects using only one element transducer having an aperture plastic mask with different coding embedded on it is used. For US scanning, a 3D object is made from plastic material consisting of alphabets “E” and “D.” Both of the letters are apart from each other at a distance of 10 mm. The size of the letters used in this work was 12 × 12 mm.

### 7.2. Simulated Datasets

Field II is a simulation software used to simulate US image and US probe along with its different attributes. In this review, we have observed that researchers have used Field II US RF simulator for experimentation and validation of their work.

In [35], a tissue mimicking phantom (model 040GSE, CIRS, Norfolk, VA, USA) B-mode image was generated using Field II. The center frequency of probe was 7.5 MHz and sampling frequency was 100 MHz. The generated image was a point phantom with a 50 dB dynamic range. Also simulated images of wire region and cyst region were generated utilizing the same settings. The point phantom consisted of six point targets with no attenuation. An RF US image of blood vessels was simulated using Field II in [36], and the size of the image was adjusted to 128 × 128 pixels. In [38], a speckle generating phantom with a hyperechoic cyst was simulated in Field II. The simulated transducer was concave probe with center frequency of 5 MHz and sampling frequency of 80 MHz.

An elastography phantom was generated using finite element analysis (FEA) and Field II in [39]. The authors have modeled a linear elastic phantom of size 40 × 50 × 10 mm^3^ having a cylindrical inclusion of D : 10 mm. The adjustment for US image acquisition was as follows: a linear probe of 152 elements, center frequency of 3.5 MHz, and sampling rate of 28 MHz. Using Field II, 128 RF lines were generated, where each line contains 2,589 samples through the depth. The phantom was designed to mimic a carcinoma in breast tissue.

A liner probe of 192 US elements is simulated using Field II simulator in [37]. The depth of transmitted and received US was focused at 70 mm. The simulated image was a phantom of 5 hypoechoic and 5 hyperechoic cysts of dimension 50 × 10 × 60 mm^3^, containing 1,000,000 scatterers. The data are calculated using delay and sum technique using continuous Hanning apodization counting all the 128 receive elements.

In [42], a modified Shepp-Logan phantom is used. The image is modified with introducing speckle noise which is presented in practical US images. This phantom is a built-in MATLAB image and is used in many biomedical image reconstructions and other image processing applications. The procedure to introduce speckle noise is as follows: the first scatterers are generated at uniformly random locations, and the amplitudes are distributed in proportion to zero-mean GGD with scale parameter set to 1 and shape parameter set to 1.3. The scatterers were further multiplied to original values of phantom pixels nearest to scatterers position. The resulting image mimics TRF. The probe settings were a 3.5 MHz linear probe, and the data were sampled in axial direction at 20 MHz. The image is blurred using a Gaussian noise of SNR 40 dBs. Field II is also used to simulate a mouse kidney and a cyst point object; both of the images were acquired by convolution of spatially invariant PSFs and TRFs. For point cyst, the same PSF settings were taken as in the previous case and TRF was a round hypoechoic inclusion with random variable, distributed according to a GGD having shape parameter set to 1. In the kidney image, the PSF was generated using Field II having central frequency of 4 MHz and axial sampling frequency of 40 MHz. A linear probe of 128 elements was used. The shape parameter of GGD was changed to 1.5 and scatterers were taken satisfactorily large enough in both cases.

In [42–45], the authors have used Field II to simulate US cyst modified Shepp-Logan and simulated mouse kidney images. In these studies, Field II is used to generate the images with different settings of PSF, TRF, and various distributions of scatterers.

## 8. Results and Discussion

Techniques of compressive sensing are continuously evolving. There has been a lot of work done by the researchers in the field of CS application to ultrasound. In this work, CS US algorithms are broadly divided into five groups.

In the first group, NMSE, NRMSE, and SSIM results are compared for different algorithms. In [32, 33], the authors used AMP and had the best results of −21.23 dB using 30 iterations of AMP framework with ABE denoiser and DCT as a sparsifier. In [34], NRMSE results of three different algorithms were compared for 33% and 50% compression ratios. The results show that IRLS-DP gives best performance in both cases. The authors in [35] have used a framework of CS-STA for different numbers of RF firings to form an US image. The advantage of CS-STA is the reconstruction of US image from less number of firing or samples, also achieving a high frame rate, high contrast resolution, and high spatial resolution for the reconstructed image. Results are compared with the original image using NRMSE. In [36], Bayesian framework was used, where the error is very small between the original and reconstructed images. Better reconstruction results can be obtained by introducing image blocking, denoising filters, or use of GPUs.

In the second group, CS recovery algorithms are discussed on the basis of different sparsifying domains. Evaluation parameters MAE, MSE, SSIM, and PSNR are used for the comparison of reconstructed data and original data. In [39] and [37], *L*_1_ minimization and stochastic sparse recovery algorithms were used. From the obtained results, it can be concluded that BSBL minimization with the wavelet atom as sparsifying matrix has the best performance. In [38], CS is applied to three different data types with DCT and DWT as sparsifying basis. Their obtained MSE results establish that CS-FLOW (CS reconstruction of RF post-beam-formed data) with a DCT model basis has minimum error. In [40], the model basis is used to calculate TPSF. In the model basis, the column coherence of beam-forming matrices has minimum off-diagonal TPSF value. The results had shown that frequency domain model matrix with Fourier sparsifying basis has more accurate PSNR and SSIM values than others. In [32], DCT, wavelet, and time domain sparsifying bases and ABE as a denoiser with the CS recovery algorithm of AMP are used. Their results confirm that DCT as a sparsifying domain and ABE as a denoiser have the best results. From all the above-mentioned research papers, it can be concluded that DCT as a sparsifying basis is the most suitable sparse basis for CS data recovery.

The third group of the review papers deals with different compression ratios for the CS of ultrasound images. This group includes algorithms which were able to achieve more accurate results at higher compression ratio up to 80%. PSNR and SSIM were calculated using the above ratios for the last two papers. The maximum PSNR and SSIM achieved are 29.29 and 80.10, respectively, for CD_true at a compression ratio of 80%. From [41], it was concluded that OMP performs faster than CoSaMP but has less accuracy in comparison. In [42], CD is used to overcome the bandwidth limitation of RF US transducer. These limitations affect spatial resolution, contrast, and SNR. In [43], a 2D convolution operator carrying system PSF information is used with sensing matrix. The framework was called compressive blind deconvolution which aims to jointly approximate system PSF and US image. In [44], a framework compressive semiblind deconvolution is used for US image recovery with an unknown PSF. The results are promising, as they have better PSF and TRF. Future direction for research is the study of parametric model of US PSF and the use of nonconvex optimization techniques. In [45], the framework of CD is used based on ADMM to recover US images. Based on the estimation of generalized distributed TRF, *L*_*P*_-norm minimization is used for values of *p* between 1 and 2. Future research scope in this area is the automatic approximation of *P* for *L*_*P*_ norm.

In group four, NRMSE was calculated for CS of 3D ultrasound. In [48], CS was introduced and applied to 3D US volumes using three different sparsifying patterns. The US volumes were reconstructed using *L*_1_ minimization with a very little information being lost at a sampling rate of 50%. In [47], to achieve sparser representation, learned overcomplete dictionaries are used. The authors used K-SVD for dictionary learning using nonlog envelop data. Results show that K-SVD with *R*_2_ sampling pattern has minimum NRMSE for all sampling ratios. In [49], K-SVD is used for sparse dictionary learning while Fourier and DCT transforms are used as sparse domain for in vivo and ex vivo data and also their results are compared. In [50], novel US image acquisition technique using sparse signal acquisition and recovery with a single element transducer and coded plastic mask is introduced. The benefits are as follows: very low cost of hardware, low power consumption, and low processing hardware for image acquisition. The future direction of research in 3D ultrasound CS is the study of different sparsifying basis and sensing matrices, optimal conditions, investigation of optimal routines or algorithms for 3D sparse recovery, sparse dictionary learning routine, and time optimization.

In the fifth group, sparse signal recovery algorithms are jointly used with DNN to improve sparse signal recovery. Some good DNN approaches to recover sparse data are reported in the literature, though a few have found their place in US image CS recovery. Also, some suggestions for improving its performance are the variation of the optimization algorithms, loss function, or new deep neural network for fast and accurate sparse signal estimation.

## 9. Conclusions

CS in medical ultrasound is a promising field of research and has opened a gateway for research on US scanners and its development. This work presents a concise review for CS in the field of US. Different modes of US image acquisition techniques for acquiring various types of preprocessed and processed data and its applications are presented. Moreover, fundamentals of ultrasound technology and its mathematical model for CS and theoretical foundations are discussed. Contributions made in various aspects of CS in US to improve image quality and to recover the US image from different types of data using various sparse bases for signal representation have also been discussed. This review also provides an overview of various algorithms and frameworks for US sparse signal and image recovery. Each acquisition technique and reconstruction framework has its own pros and cons and there is always a tradeoff between reconstructed image quality, computational complexity, sampling ratio, and noise in the image. Researchers suggested various model bases, sensing matrix, and noise removal frameworks for CS ultrasound reconstruction of sampled data at different rates, yet there is no perfect model for US image acquisition and reconstruction.

## Figures and Tables

**Table 1:** CS-based reconstruction algorithms.

S/N	References	Method	NMSE after 30 iterations				SSIM		
1	[32]	Approximate messaging passing DCT, wavelet, and spatial as transform domain Soft thresholding and ABE as denoiser	Time soft thresholding			−5 (dB)			
			Time ABE			−5 (dB)			
			Wavelet soft thresholding			−10 (dB)			
			Wavelet ABE			−10.12 (dB)			
			Discrete cosine transform ST			−13.97 (dB)			
			Discrete cosine transform ABE			−21.23 (dB)			

2	[33]	Approximate messaging passing Cauchy prior-based maximum a posteriori	Algorithm		Time (sec)				
			ST		4.57	−14.25 (dB)			
			ABE		4.77	−15.15 (dB)			
			Cauchy-MAP		5.33	−16.27 (dB)			

3	[34]	IRLS-DP FD-S*α*S-IRLS S*α*S-IRLS *L*_*P*_ minimization (DP) dual prior information	NRMSE				S*α*s-IRLS	FD-S*α*SIRLS	IRLS-DP
			Compression ratio	S*α*s-IRLS	FD-S*α*SIRLS	IRLS-DP			
			33%	0.697	0.540	0.249	0.208	0.586	0.908
			50%	0.518	0.291	0158	0.377	0.844	0.944

4	[35]	CS-STA Sym8 wavelet as sparsifier	NRMSE						
			CS-STA	32	64	128			
			Results 1	0.98%	0.42%	0.01%			
			Results 2	0.41%	0.12%	0.001%			

5	[36]	Bayesian framework-based algorithm Fourier transform as sparse domain	NRMSE						
				Simulated image		In vivo image			
			Classical CS	*E* = 0.12		*E* = 0.10			
			Bayesian CS	*E* = 0.12		*E* = 0.07			

**Table 2:** CS reconstruction algorithm based on sparsifying transforms.

S/N	Reference	Method	Average MAE					
1	[39]	Model basis (i) Wavelet atom (ii) D cosine T (iii) DFT reconstruction (iv) *L*_1_ minimization (v) BSB	L1-FT		1.8994*e* − 04			
			L1-DCT		1.3124*e* − 04			
			L1-WA		1.2161*e* − 04			
			BSBL-FT		1.3693*e* − 04			
			BSBL-DCT		9.5381*e* − 05			
			BSBL-WA		1.6805*e* − 04			

2	[37]	Model basis (i) Directional wave atoms (ii) Daubechies wavelets (iii) Fourier transform	L1-wavelet		1.5163*e* − 03			
			L1-DCT		8.3572*e* − 04			
			L1-W atom		5.5428*e* − 04			

3	[38]	Model basis (i) DWT (ii) DCT Reconstruction Convex optimization *L*_1_ minimization	MSE					
			CS-flow 1			2.34		
			CS-flow 2			2.95		
			CS-flow 3			4.34		

4	[40]	Model basis (i) curvelets (ii) Wavelet (iii) Cosine (iv) Fourier	Method	PSNR				SSIM
			*Cyst phantom image*					
			Frequency domain	25.758			0.726	
			Time domain	22.857			0.701	
			*Liver image*					
			Frequency domain	32			0.783	
			Time domain	20.2			0.741	

5	[32]	(i) Approximate messaging passing model basis (ii) DCT (iii) Wavelet (iv) Spatial domain ST and ABE as denoiser	Time ST	9.09			0.14	
			Time ABE	8.57			0.09	
			Wavelet ST	12.46			0.28	
			Wavelet ABE	12.38			0.25	
			DCT ST	18.56			0.54	
			DCT ABE	23.95			0.80	

**Table 3:** CS reconstruction algorithm based on compression ratio.

S/N	Reference	Method	PSNR					SSIM			
1	[41]	Orthogonal matching pursuit for CS Compressive sampling matching pursuit	Algorithm	20%	40%	60%	80%	20%	40%	60%	80%
			OMP	24.13	24.33	24.40	25.10				
			CoSaMP	26.44	26.48	26.48	26.75				

2	[42]	ADMM compressive deconvolution GGD	ADMM	24.77	25.28	26.03	26.82				

3	[43]	Compressive sampling image deconvolution AM-based algorithm for compressive blind deconvolution		21.48	22.59	23.12	24.39				

4	[44]	CSBD SDMM TRF and PSF	CD_true	25.29	27.07	28.57	29.29	61.07	73.91	78.14	80.10
			CD	22.72	22.49	22.33	22.32	45.76	49.66	50.51	52.04
			CSBD	25.01	26.87	27.31	28.55	58.36	73.22	77.35	80.03

5	[45]	Compressive deconvolution based on ADMM *L*_*p*_-norm minimization	Dataset	B-PSNR							
			Image 1 proposed	28.97	40.65	47.28	52.33	22.40	43.74	65.06	72.52
			Image 1 sequential	49.25	40.98	31.90	22.34	20.75	36.82	55.72	70.93
			Image 2 proposed	73.75	66.98	52.38	52.12	26.23	52.80	59.06	60.25
			Image 2 sequential	36.46	29.60	25.90	23.87	18.32	28.02	40.89	39.89

**Table 4:** CS reconstruction of 3D ultrasound.

S/N	Reference	Method	NRMSE					
1	[48]	K-SVD overcomplete dictionaries *R*_1_ and *R*_2_ sampling patterns DCT basis Fourier basis	Sampling masks *R*_1_ and *R*_2_			20% sampled	50% Sampled	80% sampled
			DCT and *R*_1_			0.59 × 10^−2^		1.31 × 10^−2^
			DCT and *R*_2_			0.54 × 10^−2^		1.35 × 10^−2^
			Fourier and *R*_1_			0.48 × 10^−2^		1.45 × 10^−2^
			Fourier and *R*_2_			0.54 × 10^−2^		1.28 × 10^−2^
			K−SVD and *R*_1_			0.31 × 10^−2^		1.06 × 10^−2^
			K−SVD and *R*_2_			0.32 × 10^−2^		0.97 × 10^−2^

2	[47]	*K*-space signal reconstruction *L*_1_ minimization routine	Sampling patterns			NRMSE results of 50% sampled data		
			Φ1			0.090 ± 4.4 × 10^−4^		
			Φ2			0.097 ± 4.4 × 10^−4^		
			Φ3			0.094 ± 20 × 10^−4^		

3	[49]	K-SVD line-wise and point-wise sampling patterns OMP for minimization	NRMSE					
					Technique	20%	50%	80%
			In vivo	Kidney	K-SVD	2.59 × 10^−4^	4.25 × 10^−4^	5.91 × 10^−4^
					Fourier	2.99 × 10^−4^	5.10 × 10^−4^	7.28 × 10^−4^
				Liver	K-SVD	2.64 × 10^−4^	4.23 × 10^−4^	5.92 × 10^−4^
					Fourier	2.98 × 10^−4^	5.07 × 10^−4^	7.24 × 10^−4^
			Ex vivo	Brain	K-SVD	2.10 × 10^−4^	3.78 × 10^−4^	5.73 × 10^−4^
					Fourier	2.51 × 10^−4^	4.67 × 10^−4^	7.85 × 10^−4^
					DCT	2.86 × 10^−4^	4.98 × 10^−4^	7.12 × 10^−4^
				Kidney	K-SVD	2.13 × 10^−4^	3.52 × 10^−4^	5.23 × 10^−4^
					Fourier	2.53 × 10^−4^	4.56 × 10^−4^	7.21 × 10^−4^
					DCT	2.75 × 10^−4^	4.83 × 10^−4^	7.29 × 10^−4^
				Heart	K-SVD	2.51 × 10^−4^	4.12 × 10^−4^	6.19 × 10^−4^
					Fourier	3.01 × 10^−4^	5.15 × 10^−4^	8.38 × 10^−4^
					DCT	3.11 × 10^−4^	5.50 × 10^−4^	8.67 × 10^−4^

4	[50]	Plastic coded mask			Results not in numerical form			

**Table 5:** CS-based deep learning novel methods.

Test case	Method	PSNR(dB)				
	Compression ratio	0.1	0.2	0.3	0.4	0.5
Carotid long [57]	SDA-L	16.03	16.49	19.60	26.10	30.43
	SDA-NL	19.20	22.73	28.33	34.11	35.57
	CS	16.27	17.74	22.98	31.73	39.24
In vitro-type 1 [57]	SDA-L	17.85	19.69	22.03	25.41	27.73
	SDA-NL	18.21	22.38	28.15	31.83	33.25
	CS	17.73	19.25	22.42	27.51	33.12

**Table 6:** Results PSNR (dB) with various sampling noise and sampling ratios.

Noises	(*α* = 1.0, *γ* = 10–3)				(*α* = 1.5, *γ* = 10–1)			
Sampling rate	20%	30%	40%	50%	20%	30%	40%	50%
LqLa-ADMM	*∗*	*∗*	*∗*	*∗*	*∗*	*∗*	*∗*	*∗*
ADMM-net	31.95	34.20	34.63	35.68	30.58	33.02	29.59	29.58
Initial-Co-robust-ADMM	27.29	29.88	30.05	30.60	27.26	29.01	30.06	30.80
Co-robust-ADMM-net	32.47	34.70	35.58	37.10	32.35	34.77	33.26	33.01
